# A dog bite study in a dog rabies-affected area in South Africa

**DOI:** 10.4102/sajid.v35i1.65

**Published:** 2020-06-22

**Authors:** Jacqueline Weyer, Chantel A. le Roux, Charles Kajese, Lucy Fernandes

**Affiliations:** 1 Centre for Emerging Zoonotic and Parasitic Diseases, National Institute for Communicable Diseases of the National Health Laboratory Service, Sandringham, South Africa; 2 School of Health Care Sciences, Department of Public Health, Sefako Makgatho Health Sciences University, Medunsa, South Africa; 3 Surveillance Information Management Unit, National Institute for Communicable Diseases of the National Health Laboratory Service, Sandringham, South Africa

**Keywords:** rabies, dog bites, rabies vaccine, rabies immunoglobulin, rabies post-exposure prophylaxis, South Africa

## Abstract

**Background:**

Rabies is an untreatable and highly fatal viral zoonosis. Despite the ability to control and prevent the disease, it is estimated that one person dies of rabies every 10 minutes in developing countries. However, the true burden of the disease remains undefined in most developing countries because of a lack of systematic surveillance. Dog bite data obtained from healthcare facilities where dog bite victims seek medical care may provide an additional source of information that can be used to inform the burden of disease and identify points for interventions for improved delivery of post-exposure prophylaxis (PEP) to prevent the disease.

**Methods:**

A descriptive epidemiological study was conducted using data obtained from dog bite registers and patient case files at a healthcare facility for a two year period (2015-2017).

**Results:**

The study reported frequency, demographics, source, geographic and temporal distribution of bite cases reported to a hospital serving a rabies-affected community. In addition, the post-exposure management of dog bite cases at this facility is described.

**Conclusion:**

Dog bites was not an infrequently reported at the healthcare facility, with up to 29 cases reported in a month during the study period. The affected population was defined and it is motivated that this information is useful for targeted health interventions. Findings related to the delivery of PEP may also be used to direct training and re-training of healthcare workers for improved delivery of PEP.

## Introduction

Rabies, a viral infection of the brain, has a near 100% fatal outcome.^1,2^ The rabies virus is transmitted to humans through the infected saliva of rabid animals, mostly domestic dogs.^2,3^ There is no effective curative therapy for rabies, and in dog rabies endemic areas, the only point of intervention is to prevent the infection through post-exposure prophylaxis (PEP).^2,3,4,5^ Rabies PEP includes thorough wound washing followed by rabies vaccination, and in exposures that have breached the skin surface, also rabies immunoglobulin (RIG) therapy.^2,6^ This treatment regimen is effective in the prevention of rabies in humans, and the so-called ‘treatment failures’ have only been noted in cases when international guidelines for rabies PEP have not been adhered to.^6,7^

Despite the availability of safe and effective rabies vaccines and immunoglobulins to protect human life, and tried-and-tested strategies of controlling the disease in dogs and other animals, rabies is still the cause of an estimated 59 000 (confidence interval [CI], 25 000–159 000) human deaths and more than 3.7 million disability-adjusted life years (CI, 1.6–10.4 million) per year globally.^8^ The brunt of rabies is experienced in the developing countries of Africa and Asia where dog rabies has not yet been controlled.^8,9^ Health service inequalities remain at the heart of the rabies problem, with poorer and typically rural communities not enjoying good access to veterinary and human health services being the most vulnerable.^8,10,11,12^ Further compounding the problem is the lack of accurate surveillance statistics that could bolster the support for rabies control and prevention programmes in the affected country.^11,12,13,14^ Apart from the loss of human life, the cost of the provision of rabies PEP to prevent rabies infection in humans where dog rabies endemically occurs presents a significant economic burden.^8,12^ The global annual expenditure for rabies PEP biologicals and delivery of the treatment has been estimated to be $8.6 billion.^8^

A global call for the elimination of dog-transmitted human rabies by 2030 has been made by the World Health Organization (WHO), the World Organization for Animal Health, the Food and Agriculture Organization of the United Nations and the Global Alliance for Rabies Control.^2^ In heeding this call, countries are required to prioritise dog vaccination programmes, improve on the provision of PEP and invest in enhanced surveillance for animal and human rabies cases. Surveillance for human rabies is complicated and not easily achieved by either an active or a passive approach.^9,15^ The frequency of the disease occurrence and the disperse nature of its geographic spread render active surveillance approaches for human cases impractical. Likewise, passive surveillance relies on astute clinical recognition of a case and specialised and complicated ante-mortem or post-mortem laboratory confirmation.^16^ The latter requires the collection of brain tissue, which is not easily attained because of various cultural, religious and increasingly operational reasons.^16,17^ Alternative surveillance approaches that can supplement the understanding of the public health burden of this disease are encouraged.^10^ One such approach is the use of dog bite surveys. Dog bite surveys have been used to report on the epidemiology of dog bites, to determine the knowledge and practices (including adherence) for rabies PEP and to provide data for the estimation of human rabies cases.^18,19,20,21,22,23,24,25^

In South Africa, active transmission cycles of the rabies virus in domestic dogs have been reported in the mid-20th century.^26^ The history of dog rabies control in South Africa is characterised by intermittent success and also failures resulting in outbreaks and consequently also human cases.^18,26^ Since 2004, the disease has emerged in locations where it was controlled before, as is evident by outbreaks in Vhembe district of Limpopo province, Ehlanzeni district of Mpumalanga province, the Bojanala Platinum district of North West province and the Botshabelo surroundings in Free State province.^27,28,29,30^ In addition, a great number of outbreaks of dog rabies were reported in Soweto, Gauteng province, in 2010, marking the first report of sustained dog-to-dog transmission of the rabies virus in this province.^31^ The locations described here are mostly densely populated, which further raises the concerns of the possibility of the human rabies cases occurring considering the close association of dog rabies outbreaks and human cases.^17,26,27,31^ On average, 10 cases of human rabies are laboratory confirmed in South Africa annually since 1983 (National Institute for Communicable Disease South Africa, pers. comm, 2019). The surveillance of human rabies in South Africa relies on the submission of samples of clinically suspected cases followed by laboratory investigation and confirmation. These statistics are considered an underestimation given the burden of dog rabies in the country.^8^ Dog bite registers are routinely kept at healthcare facilities in South Africa, although dog bites are not considered a notifiable medical condition. However, the data collected in the registers are not routinely analysed in order to monitor the epidemiology of dog bite cases or used to direct public health responses. In this study, data obtained from bite registers from a hospital serving a community affected by dog rabies in Mpumalanga province of South Africa were investigated.

## Materials and methods

### The study population and setting

The study was based at the Mapulaneng Hospital, which is a public healthcare facility serving the community of Bushbuckridge Local Municipality, Mpumalanga province, South Africa. According to the 2011 census, Bushbuckridge has a population of 541 248 people and 134 197 households, rendering it the seventh biggest municipality in South Africa.^32^ The population comprises 99.5% black Africans, of which 52.1% of the economically active population were unemployed.^32^ The setting is primarily rural, with nearly 40% of the households employing pastoral farming.^32^ In addition to the Mapulaneng Hospital, Bushbuckridge is served by two other hospitals, five community health centres and 34 primary healthcare clinics. The study population included all animal bite cases registered at the Mapulaneng Hospital for the period August 2015–July 2017.

### Data collection

A retrospective document review was performed, using hard copy dog bite registers as the source of data. The data were captured in a Microsoft^®^ Excel spreadsheet. Data for the following variables were collected: sex, age in years, geographic location and animal involved in exposure and date of hospital visit. To study the PEP management of patients, the patient files for cases noted in the dog bite register for the 6 month period (January–July 2017) were considered. The PEP data were collected using a data extraction tool which was designed in EpiInfo™ 7.2.1.0 software. The data extraction tool included 19 questions and a free text field for any additional information to be captured. The tool comprised three parts: patient demographics, animal bite details and PEP management details. The contents of the data extraction tool were based on the ‘Record form for possible exposure to rabies as provided by the WHO Expert Committee on Rabies’ (WHO, 2013).

### Data analysis

The data from the bite registers were collected in a Microsoft^®^ Excel spreadsheet. Graphs were constructed in Microsoft^®^ Excel. Basic descriptive statistics were summarised by using Stata^®^ 13.0 software. Density and distribution mapping using approximate global positioning system coordinates for geographical location of exposure was performed in ArcMap mapping software. Data from the patient files were collected using an EpiInfo^®^ designed data collection tool. The data captured in EpiInfo^®^ were automatically used to populate a Microsoft^®^ Access database.

### Ethical considerations

The research protocol was reviewed by the Sefako Makgatho Health Sciences University School Research Ethics Committee followed by approval from the Sefako Makgatho Health Sciences University Research Ethics Committee (reference number: SMUREC/H/10/2017: PG). Permission for the study was then obtained from the Chief Executive Officer of the Provincial Department of Health, Mpumalanga, followed by approval from the Provincial Health Research and Ethics Committee of Mpumalanga Province (reference number: MP_2017RP46-339).

## Results

### Occurrence of bite cases

**FIGURE 1:**
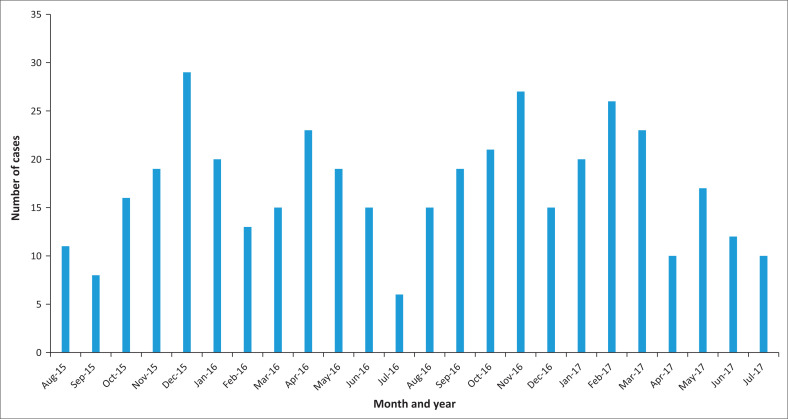
Number of animal bite cases per month presenting to Mapulaneng Hospital for management, 01 August 2015–31 July 2017 (*n* = 411).

A total of 411 dog bite cases were reported for the study period, relating to an average of 17cases (range: 6–29) per month (Figure 1). A total of 194 cases were reported during the first year of study, with an increase to 215 cases for the second year of the study. Although clear and consistent seasonality was not observed, more cases were noted for both study years during summer months. The reports almost exclusively indicated the involvement of domestic dogs as the animal type linked to the cases (*n* = 407, 99.0%). Two cases of exposures to domestic cats, a pig and a monkey, were also reported (results not shown).

**FIGURE 2:**
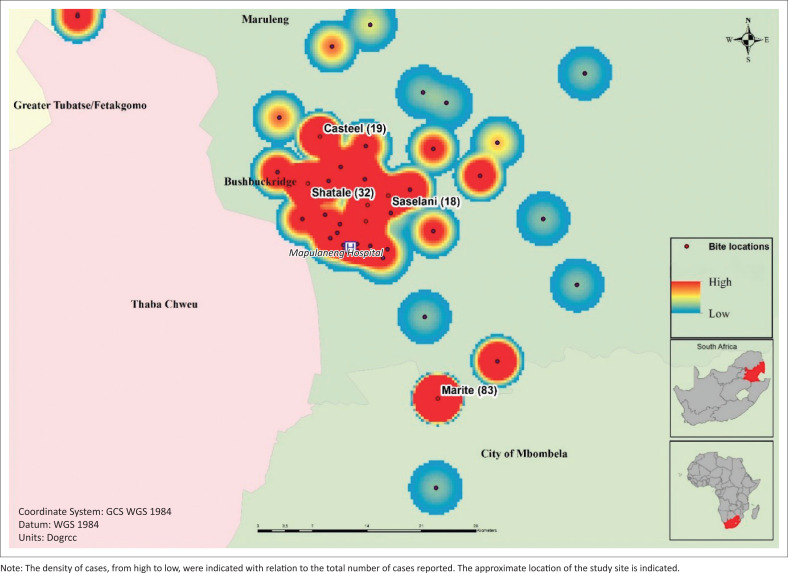
Density distribution of dog bite cases in this study.

A density map of the dog bite cases indicated the geographical distribution of the cases, identifying several hotspots for dog bites (Figure 2). The geographical location of the dog bite exposures was available for all but one of the cases reported. Nearly one-fifth (*n* = 82) of the cases were reported from Marite, located about 20 kilometre (km) southeast of the hospital. Marite has a population of 8657 according to the 2011 census.^32^ The incidence of dog bite cases from Marite, presenting to Mapulaneng Hospital for management during the study period, was therefore 9 per 1000 population. Secondly, nearly 8% of the cases were reported from Shatale. Shatale is similar in size as Marite, with a population of 9155 as per 2011 census.^32^ A total of 32 dog bite cases from Shatale presented to Mapulaneng Hospital during the study period, resulting in an incidence of 4 per 1000 population for the study period. The incidence of dog bite cases in these locations would represent an underestimate number, given that not all dog bite cases would report to a healthcare facility for treatment, and other public health facilities are available in Bushbuckridge.

### Demography of dog bite cases

**FIGURE 3:**
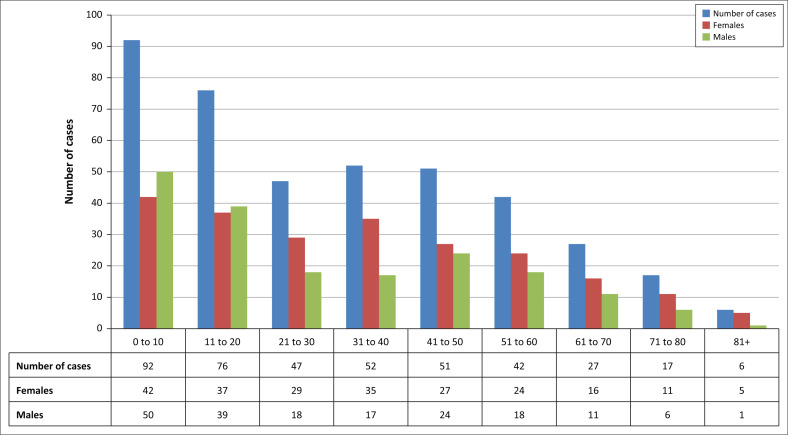
Gender and age distribution of dog bite patients reporting to the Mapulaneng Hospital, 01 August 2015 – 31 July 2017 (*n* = 410, one person’s age was not recorded).

More than half of the dog bite case victims were women (*n* = 227, 55.23%) (Figure 3). The age of one of the patients studied was not recorded, and the age of patients ranged from 0.5 to 89 years, with a mean age of 31 years. The age distribution of patients was skewed towards younger individuals. Nearly a quarter of the cases were reported in children under the age of 10 years, and 40% of the cases reported in under 20-year-olds.

### Management of dog bite cases

#### Characteristics of bite cases reviewed for post-exposure management

A total of 53 patient files were available for data extraction to analyse the delivery of rabies PEP for the period January–July 2017. The patients comprised 31 women and 22 men aged between 6 and 83 years (mean age 34.02 years), with two-thirds of the cases reported in under 20-years-olds. Of the 53 cases reviewed, 51 cases were linked to dog bites, with an additional two domestic cat exposures reported. One of the records reviewed referred to a snake bite in May 2017 for which rabies vaccination was prescribed (not recorded in the dog bite register). The same patient then suffered a dog bite in June 2017 for which the case was recorded in the register and rabies PEP was repeated.

Only two of the case files indicated that the exposures were either provoked or unprovoked, and in more than 90% of the documents no note was made whether the dog was a stray or an owned dog. In all but three of the cases, the exposures were categorised. The majority of exposures were deemed to be category 2 exposures (*n* = 39, 73.58%), which, according to the WHO guidelines, imply potential exposure on intact skin requiring wound treatment as needed and rabies vaccination. Only one exposure was deemed as a category 3 exposure, which would require for full rabies PEP (i.e. including RIG). A description of the wounds was available for 19 of the 53 patients. Based on these descriptions, a review of the categorisation of the exposure was performed. The snake bite case was managed as a category 3 rabies exposure but should have been managed as a category 1 exposure as snakes are not considered a risk for rabies virus exposure. Of the remaining cases, 16 (84.21%) were deemed to be category 3 exposures. Description of the wounds, for example, as ‘bleeding’ or ‘requiring suturing’, was interpreted as wounds that would qualify as category 3 exposures. Two cases that were originally categorised as category 1 but indicated the presence of abrasions were reviewed as category 2 exposures.

#### Details of post-exposure management

**Wound treatment:** Nearly all patients reviewed received prescriptions for antibiotic treatment to address possible bacterial infections of the wounds (*n* = 47, 90.38%). Reports of the provision of tetanus vaccination were available for 30 of the 53 patients, whilst 18 of the patients had an indication of only wound washing being performed.

**Rabies vaccination and immunoglobulin treatment:** Following the review of cases, it was noted that the hospital typically provides initial vaccination and RIG treatment to patients, after which the patients are referred to local clinics for follow-up vaccination (*n* = 51, 96.23%). Some patients were referred to the hospital for RIG only. Only two of the patients reviewed returned to the hospital for a follow-up vaccination as opposed to presenting to a local clinic. For the 53 patients reviewed, contact details were available on file for 42 (79.25%) of the patients, but not traceable for 11 (20.75%) of the patients.

A total of 15 records indicated the prescription of the rabies vaccine regimen for follow-up. Of these, 10 prescriptions indicated referral for the five-dose intramuscular schedule of rabies vaccination and only one for the currently recommended four-dose intramuscular schedule. Other variations of regimen prescribed included a three-dose and six-dose schedule. Records of RIG treatment were available for 46 of the 53 patients reviewed. One patient was referred because of the unavailability of RIG stock at the hospital.

For five of the patients reviewed (9.43%), RIG was partially administered in the gluteal muscle. It was also noted for one of the patients that RIG was administered despite a history of recent rabies vaccination administration.

## Discussion

This study aimed to consider the data that could be extracted from dog bite registers, which are kept routinely at hospitals in South Africa and the potential use of such data at the hand of simple descriptive analyses. Data were available to characterise the ‘what’, ‘who’, ‘where’ and ‘when’ questions associated with dog bites in the study area. In order to evaluate the PEP management at the study site, patient files for a 6 month period were reviewed.

Dog bites were frequently reported at the study site, with up to 29 cases reported in a month during the study period. Firstly, nearly all cases were associated with dog exposures, with only two cases of cat bites and one pig bite reported during the study period. No cases of exposures to wildlife were reported although the study setting is rural and in close proximity to wildlife reserves. Interestingly, upon review of patient files, it was noted that a patient of snake bite was also treated with rabies PEP although that case was not recorded in the dog bite register. Snake bites do not pose a rabies risk. It would be recommended that rabies risk reduction efforts should include awareness and education of particularly dog bite prevention in the affected communities.

The target population for interventions was further defined by the indication that more young women were affected by dog bites. This finding is in contrast with what has been generally observed before, that is, that men are proportionally more affected than women.^18,19,20^ The reasoning for this difference was not investigated in this study. Importantly, it is an indication for the need to analyse site-specific data to determine the risk groups and their characteristics which should not be universally accepted based on studies involving other study populations. Rabies almost universally affects children more than any other age group, as supported in this study.^8^

Although dog bites were geographically distributed throughout the Bushbuckridge municipality, a few hotspots were identified for the study period. These locations (be it villages, informal settlements, etc.) could be the targets for health awareness and education campaigns, not only for the reduction of dog bites but also to inform on the risk of rabies. This information should also be useful to veterinary services and dog rabies vaccination campaigns strategised in such a way that these areas with higher risk for dog bite are suitably covered.

Although the study was only performed using data collected over a 2-year period, it appeared that there may be seasonality associated data with the dog bite cases. More cases were reported during the summer months of October to January. This observation was also made in other studies involving communities in locations around the world.^33,34,35^ Dwyer and co-workers^36^ also found an increased number of dog bite cases reported in Cape Town, South Africa, during the summer months of November to January. The trend is generally ascribed to factors such as the different social behaviours of humans during the summer months (i.e. children playing outdoors more, the longer school holidays, etc.). The seasonality of rabies in South Africa has been recorded as during the late winter and early spring months of July to September.^26^ This inference was however based on data collected prior to 1994 and an updated study in this regard has not been published to date. In Zimbabwe, no significant rabies seasonality was noted, although a slight increase of cases was reported for the months June to November.^37^ The same was found in a study in Namibia.^38^ Again, this information could be useful in the strategic design of awareness and education campaigns for this community.

Several points of deviation from the national and international guidelines for rabies PEP were noted. Wound washing is considered an important component of effective rabies PEP as it serves to physically remove the virus from the point of entry into the body.^6^ Wound treatment with wound washing was reported for only one-third of the cases. It is however likely that this form of treatment was performed but not recorded on the patient files. Tetanus vaccination and antibiotics, which are general components of animal bite wound care, were also often provided. For the administration of RIG, the guidelines hinge on the categorisation of exposures.^6^ Category 1 exposures include interactions with rabid or potentially rabid animals, but did not include contact with infectious saliva of the animal (e.g. petting such an animal).^6^ For category 2 exposures, which imply wounds that do not break the skin, only vaccination is required, whilst for category 3 exposures, both the vaccine and RIG therapy are recommended. For the cases investigated, rabies vaccine and RIG were provided regardless of the category of exposures noted on the patient records. For example, nearly 20% of cases were categorised as category 1 exposures, which would require no intervention, yet rabies PEP was provided fully. Most cases were reported as category 2 (73.58%) which would only require the administration of vaccine, yet RIG was provided in most cases. A review of the description of the exposures allowed for the re-categorisation of the exposures for 19 of the cases under investigation. Most of the exposures could be re-categorised as category 3, which would require the full rabies PEP provided.

The hospital provides referral services, that is, patients are often referred to the hospital for RIG, or receive initial rabies vaccine and RIG and then referred to clinics for follow-up vaccination. It was therefore not possible to fully determine the level of adherence of patients for follow-up rabies vaccination. The vaccine regimens prescribed for 15 patients were available and indicated that only one of the prescribed regimens was in line with the recommended four-dose intramuscular schedule (for days 0, 3, 7 and 14). The majority of patients received prescriptions for the outdated five-dose schedule (*n* = 10, 66.66%). A second finding indicated the administration of RIG in the gluteal muscle in nearly 10% of patients reviewed. This practice is strongly discouraged because administration of RIG in the gluteal muscle does not allow for sufficient absorption of the product, thus rendering it less effective.^6,10^ One particular patient was noteworthy. This patient was treated with full rabies PEP following a snake bite, and then 3 months later provided again with full rabies PEP following a dog bite. Firstly, rabies is not transmitted by snakes whatsoever and therefore rabies PEP is not indicated in the treatment of snake bites. Secondly, following rabies vaccination, the usefulness and indication of the use of RIG falls away for any subsequent exposures. The patient could be managed in line with the prescribed guidelines, through the provision of booster rabies vaccination only.

## Conclusion

This study provides a descriptive analysis of the epidemiology and PEP management of dog bite cases reporting to a hospital serving a community affected by dog rabies. This information could be useful both for the design of interventions for the prevention of transmission at the community level and to identify challenges in delivery of appropriate PEP. It is recommended that a programme for the systematic collation and analysis of dog bite data at facility, district and provincial levels would contribute to the aim of the reduction of human risk, and ultimately the eradication of dog-transmitted human rabies by 2030.
